# The Generic Risks and the Potential of SDN-1 Applications in Crop Plants

**DOI:** 10.3390/plants10112259

**Published:** 2021-10-22

**Authors:** Katharina Kawall

**Affiliations:** Fachstelle Gentechnik und Umwelt, Frohschammerstr. 14, 80807 Munich, Germany; info@fachstelle-gentechnik-umwelt.de

**Keywords:** genome editing, CRISPR/Cas, site-directed nuclease, SDN-1, risk assessment

## Abstract

The use of site-directed nucleases (SDNs) in crop plants to alter market-oriented traits is expanding rapidly. At the same time, there is an on-going debate around the safety and regulation of crops altered with the site-directed nuclease 1 (SDN-1) technology. SDN-1 applications can be used to induce a variety of genetic alterations ranging from fairly ‘simple’ genetic alterations to complex changes in plant genomes using, for example, multiplexing approaches. The resulting plants can contain modified alleles and associated traits, which are either known or unknown in conventionally bred plants. The European Commission recently published a study on new genomic techniques suggesting an adaption of the current GMO legislation by emphasizing that targeted mutagenesis techniques can produce genomic alterations that can also be obtained by natural mutations or conventional breeding techniques. This review highlights the need for a case-specific risk assessment of crop plants derived from SDN-1 applications considering both the characteristics of the product and the process to ensure a high level of protection of human and animal health and the environment. The published literature on so-called market-oriented traits in crop plants altered with SDN-1 applications is analyzed here to determine the types of SDN-1 application in plants, and to reflect upon the complexity and the naturalness of such products. Furthermore, it demonstrates the potential of SDN-1 applications to induce complex alterations in plant genomes that are relevant to generic SDN-associated risks. In summary, it was found that nearly half of plants with so-called market-oriented traits contain complex genomic alterations induced by SDN-1 applications, which may also pose new types of risks. It further underscores the need for data on both the process and the end-product for a case-by-case risk assessment of plants derived from SDN-1 applications.

## 1. Introduction

Genome editing encompasses a suite of different techniques, which include, but are not limited to, site-directed nucleases (SDNs) and oligonucleotide-directed mutagenesis (ODM) [1]. The main focus in this paper is on SDNs, which include zinc finger nucleases (ZFNs), transcription activator-like effector nucleases (TALENs), meganucleases (MNs), or ‘clustered regularly interspaced palindromic repeats/CRISPR associated protein’ (CRISPR/Cas). SDNs have increased the possibilities worldwide for researchers and breeders to alter the genome of target organisms in a way that is extremely difficult or impossible to achieve using conventional breeding techniques. Conventional breeding relies primarily on crossing and selection of offspring with the desired characteristics, sometimes combined with induced non-targeted mutations. SDNs have already been used in many organisms and cell lines, including humans, animals, plants, fungi, and bacteria [2,3,4,5,6,7,8]. ZFNs, TALENs, and meganucleases are protein-based systems using chimeric nucleases composed of sequence-specific DNA-binding molecules linked to a non-specific DNA cleavage domain (e.g., FokI) [9,10,11]. CRISPR/Cas is a RNA-guided nuclease that has, over the last eight years, become one of the most frequently used SDN techniques in plants [12,13]. CRISPR/Cas is a versatile tool that is cheaper, simpler, and more flexible to use compared to the other SDNs. The most prominent Cas nuclease is Cas9 from *Streptococcus pyogenes*, but there are also other Cas proteins from different bacterial origins [14]. The Cas nuclease is directed to a specific region of the genome by a guide RNA (gRNA) where it creates a DNA double-strand break (DSB) upon target site recognition [15,16,17].

All SDNs can achieve genetic alterations at specific, predefined target sites through induced DSBs. DSBs activate the cell’s own repair mechanisms, resulting either in the original or an altered DNA sequence at the target site. The DSBs are either repaired by non-homologous end joining (NHEJ) or homology-directed repair (HDR). The NHEJ pathway is known to be error-prone and can result in undirected base insertions or deletions (indels), or base substitutions at the DSB [18]. In cases where the target site is altered and not restored, the reading frame of the target gene may have shifted [19]. SDNs that induce small-sized, undirected alterations at the target site are called site-directed nuclease-1 (SDN-1). DSBs can also activate HDR, thus causing sequence replacements at the target site(s) through homologous recombination using DNA templates. DNA templates consist of DNA sequences that are homologous to the targeted DNA sequence. When DNA templates are introduced into the plant cells, the target site can be altered intentionally with either a few base pairs, known as an SDN-2 application, or longer DNA sequences, known as an SDN-3 application [5,20,21,22]. An application is defined as the usage of a certain technique (e.g., CRISPR/Cas or, more generally, SDNs). SDN-1 applications are used mainly in plants cells as the NHEJ mechanisms repair DSBs more efficiently than HDR [13,23,24].

Recently, a technology known as base editing has been developed. This technology uses a catalytically inactive “dead” Cas9 variant (dCas9) coupled to cytosine or adenosine deaminase domains [25,26]. In base editing, dCas9 recognizes the target site, but, instead of inducing a DSB there, it introduces single nucleotide changes from C to T or from A to G.

In 2018, the European Court of Justice ruled that genome-edited organisms are regulated under the full provisions of Directive 2001/18/EC for the deliberate release of genetically modified organisms (GMOs) [27,28]. Thus, in the EU, all genetically engineered organisms, including plants altered by SDNs, need to undergo an environmental as well as food and feed risk assessment [20]. The European Commission (EC) recently published a study on the status of new genomic techniques (NGTs), which also includes SDNs [29]. The study concluded that current GMO legislation is not fit for purpose in regards to the regulation of genome-edited plants and needs revision. The study does not provide concrete policy recommendations, but the EC has now initiated a consultation process to discuss a new legal framework for NGTs. A number of stakeholders believe that some SDN applications should be exempt from regulation [30,31], while others are in favor of retaining the current regulation [12,32,33].

In the discussion on the regulation of NGTs, products resulting from SDN-1 applications are, in particular, frequently presented as being indistinguishable from naturally occurring mutations or alterations induced using conventional breeding techniques. However, this does not apply to all SDN-1 products. Many SDN-1 applications induce changes that go beyond the reach, possibilities, and speed of conventional methods [34,35,36]. SDN-1 can, for example, be used to alter multiple gene copies or different genes simultaneously. Furthermore, their mode-of-action increases the possibility of inducing mutations in genomic regions that are naturally correlated with the occurrence of fewer mutations [37,38,39]. The overall aim of this review was to investigate how SDN-1 applications are used to alter crop plants and the extent of their complexity. The complexity referred to here is the combination of several alterations that might be unlikely to occur naturally or achieved with random mutagenesis. It also includes an analysis of market-orientated SDN-1 applications in crop plants and determines the proportions of specific SDN-1 application types. SDN-1 applications were divided into the categories ‘single gene knockout’, ‘multiple gene copies’, and ‘multiplexing’. Both the characteristics of the genome of the respective crop plants and the types of SDN-1 application were considered in the categorization. The review further includes a discussion on the implications of the findings for SDN-1 applications in regard to product-based risks.

It further summarizes generic and new types of risks associated with SDN-1 applications which should be considered in risk assessment. Overall, a case-specific risk assessment considering both process- and product-based risks seems to be best suited to analyze plants derived from SDN-1 applications.

## 2. Advantages of SDNs over Conventional Breeding

SDNs have advantages over conventional breeding techniques, as they are faster and more targeted. This enables plant breeders to handle some major obstacles of plant genomics. Plant genomes are diverse with respect to their size, ploidy level, and heterozygosity and they contain highly repetitive genomic regions. In addition, many plant traits are determined by multiple genes and some regions of the genome are extremely difficult to access, thus posing serious challenges for plant breeders and scientists.

Many major crop plants are polyploid, i.e., they contain more than two paired sets of chromosomes which either originate from the same (i.e., autopolyploid(s)) or related species (i.e., allopolyploid(s)) [40]. In allopolyploid plants, several homoeologs contribute independently to a phenotype. Induced random mutagenesis, which is carried out via irradiation or treatment with chemicals to produce random mutations, only generates plants with single mutations in one or the other homoeolog. The probability of double mutations occurring in specific genomic regions is extremely low. Different mutations need to be combined in one genotype through time-consuming crossing and backcrossing procedures. SDNs are able to target multiple gene copies or gene variants (e.g., alleles) simultaneously, which is especially beneficial in polyploids. SDNs can also be specifically designed to target the respective nuclease towards individual alleles.

Plants have further genetic restrictions which complicate conventional breeding. For example, they have a high level of heterozygosity, often contain repetitive genomic regions due to transposable elements, and some of their genes are located in peri-centromeric regions. The local recombination rate around some genes can be suppressed in certain genomic regions, for instance, in peri-centromeric regions [41,42]. Using CRISPR/Cas makes it possible to circumvent these genetic restrictions by, for example, rearrangements of the target genes [43], by direct gene targeting, or by deletion of large genomic regions.

Plant traits are often controlled by multiple genes, for example, for abiotic or biotic stress tolerance. Generating gene knockouts of multiple genes is a time-consuming process in plant breeding and in many cases not achievable. CRISPR/Cas can be used in an application type called multiplexing, which can be employed to alter multiple target genes simultaneously by using different gRNAs. A prerequisite for multiplexing (and genome editing in general) is that the genes responsible for a certain trait and their DNA sequence are known. This is supported by the fast progression of genomics in the last decade.

Evolutionary biology theory assumed that the mutation rate is unbiased, i.e., that it varies randomly among genomic loci irrespective of the fitness consequence, and that the establishment of mutations is strongly influenced by selection [44]. However, more recent findings challenge the assumption of randomness. It was shown that mutation rates in *Arabidopsis thaliana* are influenced by cytogenetic features and that the probability of de novo mutations is dependent on gene function and fitness consequences [37,38]. Previous studies in, for example, *E. coli* and human cancer cells, already indicated a mutational bias which correlates with the DNA sequence, epigenetic features and various DNA repair mechanisms [39,45,46,47,48,49,50,51,52,53,54]. Work done in *A. thaliana* by Belfield et al. [38] showed that the DNA mismatch repair (MMR) system preferentially protects specific genic parts of the genome from new mutations. Monroe et al. [37] analyzed data of spontaneously occurring de novo mutations in *A. thaliana* and showed that mutation rates across the genome are associated with different cytogenetic features, including the GC content (i.e., guanine-cytosine content), DNA methylation, histone modifications, chromatin accessibility, gene expression, and the presence of the MMR system depending on the genomic region. These findings indicate that cytogenetic features differentially direct the DNA mismatch repair components to functionally-constrained genes most likely via binding of the histone modification H3K36me3 [37], which had already been discussed in previous studies [38,45,55,56]. Thus, mutations occur more frequently in some parts of the genome in *A. thaliana* than in others. SDN applications allow efficient introduction of targeted mutations in coding regions that are naturally associated with lower mutation rates. The ability of SDNs to recognize the target site(s) and to repeatedly induce a DSB, should the original state be restored by repair mechanisms, is a key difference between SDNs and naturally occurring or otherwise induced mutations.

Thus, SDNs are highly powerful tools that can be used in a wide range of applications to alter plant genomes in a way that is hardly achievable when using conventional breeding techniques.

## 3. SDN-1 Application Types in Crop Plants

The aforementioned potential and advantages of SDNs over conventional breeding already indicated the wide range of applications of SDNs in plants. Until now, there has been insufficient comprehensive analysis of the complexity of SDN-1 application types in market-oriented plant studies. This review reinvestigates market-oriented SDN-1 applications in crop plants derived from the dataset generated by Modrzejewski et al. [57,58] and determines the proportion of three different SDN-1 application types. It firstly describes the criteria used by Modrzejewski and coworkers to generate the dataset of market-oriented applications in plants [13], and subsequently describes how these studies were assigned to three different categories of SDN-1 application types. The review also presents the outcomes of the categorization.

### 3.1. Data Collection of Market-Oriented Applications of Genome Editing Techniques in Crop Plants

This current paper is based on the dataset generated by Modrzejewski et al. [13] who conducted a comprehensive systematic literature screen in order to analyze the applied genome editing techniques and edited plant species between January 1996 and May 2018. In their dataset, the authors differentiated between applications in basic research and market-oriented genome editing applications. They applied the following three criteria that need to be fulfilled in order to categorize a study as market-orientated:

1. A genome editing technique (including CRISPR/Cas9, ZFN, TALENs, meganucleases, ODM or base editing) was applied in a crop plant;
2. A trait was edited that may be of interest for commercialization (market-oriented trait);
3. The targeted trait is expressed in the resulting genome edited plant grown.

An update of their work is available, focusing on market-oriented applications of genome editing techniques in crops and ornamentals between 1996-June 2019 [58]. In total, they identified 217 publications comprising 231 market-oriented studies [58,59]. A publication can contain several studies, i.e., different genome editing techniques, crops, or target genes are considered and listed individually. The majority of the studies used CRISPR/Cas, more specifically, mostly for SDN-1 applications in crops. A detailed summary of their findings can be found elsewhere [13,58,59].

There are caveats that need to be clarified in regard to the interpretation of the results obtained by Modrzejewski et al. [13,58]: despite the classification as “market-oriented”, much of the research done in these studies is still preliminary and there is no indication of whether the plants will be placed on the market soon or at all. Presently, only two genome edited crops are being sold on the market: soybean plants in the US, altered using TALENs, with a changed fatty acid composition [60] and tomato plants in Japan that were altered using CRISPR/Cas9, with an increased GABA-content [61,62].

### 3.2. Methodology for the Categorization of SDN-1 Application Types

**Figure 1:**
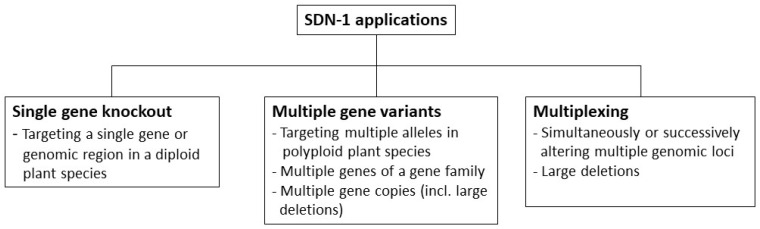
Characteristics of three different categories of genetic variations induced by SDN-1 applications. SDN-1 applications were assigned to one of the three categories ‘single gene knockout’, ‘multiple gene variants’, and ‘multiplexing’ when one of the listed characteristics was met.

Based on the dataset generated by Modrzejewski et al. [58], market-oriented SDN-1 applications in crops were analyzed and assigned to three different categories, namely ‘single gene knockout’, ‘multiple gene variants’, and ‘multiplexing’ (Figure 1). These categories are based on the specific SDN-1 application. Multiple alterations in a plant induced by SDN-1 were assessed in combination, but not in isolation. Applications of SDN-2, SDN-3, base editing, and ODM investigated in the dataset of Modrzejewski et al. [58] were not included in the analysis presented in this paper. Since the results of some studies could not be assigned to only one category, they are listed in multiple categories [42,63,64,65].

#### 3.2.1. Single Gene Knockout

A study was classified as a ‘single gene knockout’ if a single gene or genomic region in a diploid plant species was targeted by SDN-1 applications inducing undirected alterations at the target site. In most studies, SDNs induce mono- or biallelic changes at the target site, ultimately resulting in single gene knockouts after regeneration and further breeding steps. Crops that are polyploid and altered in multiple or all copies of a particular gene region, were not considered to be a ‘single gene knockout’, but are listed under ‘multiple gene copies’. The category ‘single gene knockout’ contains small deletions at the respective target site, as well as small targeted deletions generated by using, for example, two gRNAs in a single gene [66,67]. This can alter the reading frame of a particular gene or impact regulatory gene regions [68]. Alterations induced to bypass genetic linkage of specific traits in diploid species were categorized as a ‘single gene knockout’ [69,70,71]. The linkage drag is, for example, relevant in tomatoes, as it affects a large part of the genome [72]. One study used CRISPR/Cas9 in order to induce a targeted recombination of homologous chromosomes in tomatoes, resulting in the formation of yellow fruits [73], and which could also be used to segregate undesirable genetic linkages.

#### 3.2.2. Multiple Gene Variants

This paper lists studies in the category ‘multiple gene variants’ if more than two genomic loci containing the same DNA sequence were altered by genome editing using a single gRNA [74] or a single pair of TALEN [75], for example, to alter multiple alleles of a gene in a polyploid crop [76,77,78], multiple members of a gene family [79,80,81], or multiple gene copies [82,83,84]. Large deletions, which were generated in order to delete multiple gene copies simultaneously, were assigned to the category ’multiple gene variants‘. Furthermore, mutations in individual gene homoeologs and their combinations generated using SDN-1 to evaluate the effects of gene dosage on specific traits in polyploid crops, were also listed in this category [42,85,86,87,88].

#### 3.2.3. Multiplexing

A study was listed in the ‘multiplexing’ category if two or more gRNAs, TALENs, or ZNFs were used to target several different genomic regions. Multiplexing can be applied simultaneously either in one experimental setup [89,90,91,92] or successively [93,94]. Where a small deletion of a particular gene was generated by using, for example, multiple, different gRNAs in a diploid plant species, that application was listed as a ‘single gene knockout’, and in a polyploid species listed under ‘multiple gene variants’ [95,96,97]. If a larger deletion was induced in order to alter different genes using multiple gRNAs, the respective studies were assigned to the category ‘multiplexing’ [98,99,100].

### 3.3. Categorization of SDN-1 Application Types

Categorization outcomes of SDN-1 application types obtained from the analysis in this paper are summarized for market-oriented traits in crop plants (Table 1). In total, 55% of all studies analyzed are ‘single gene knockouts’, whereas 27.5% altered ‘multiple gene variants’ and 17.5% used ‘multiplexing’. Furthermore, it details the quantity of the SDN-1 application types per year. Overall, there is a trend towards the development of more complex SDN-1 induced alterations.

#### 3.3.1. Results of the SDN-1 Application Type Categorization

There are only a few market-oriented studies which used SDNs in plants for increased **tolerance to abiotic stress**. The categorization of SDN-1 application types shows that three out of six studies induced ‘single gene knockouts’, whereas two studies fall into the category ‘multiple gene variants’ and one study into ‘multiplexing’ (Table 1). Plants, planting systems, and farming systems with increased resistance and resilience to abiotic stress are urgently needed to deal with the challenges of climate change [101], but the complexity of stress-responses in plants is reflected in the small amount of publications in this category. Some studies show the difficulty of generating plants tolerant to abiotic stress, since the opposite to the intended effect was observed [102], or the stress response of the genome edited plants was not even tested under stress conditions [68,103], or only a mild tendency to drought resistance [104] was observed. There appears to be one promising study in this category. The study used CRISPR/Cas to knock out a gene in rice which resulted in significantly enhanced salinity tolerance under greenhouse conditions [105]. How the genome edited rice would respond to salinity stress under field conditions is still unclear.

Several market-oriented studies used SDN-1 in plants with the aim of increasing **resistance to biotic stress**, including viral, fungal, and bacterial infections. In total, 15 studies fall under the category ‘single gene knockouts’ (see Table 1), and most of these studies were performed on diploid rice and tomato plants. The target regions in that category are often promoter regions of host susceptibility genes within the plants, and the intention was to increase resistance to TALE-dependent pathogens [106,107,108]. Other studies targeted regulators of different signaling pathways, such as the brassinosteroid, jasmonic acid, or salicyclic acid signaling pathways [109,110] as well as different plant transcription factor families [110,111,112,113], which are all involved in multiple processes in plants. Most studies (61 of 178) fall into the category **altered agronomic value** (Table 1) and were mainly performed with rice and tomato plants. Over half (35) used ‘single gene knockout’ and a little less used ‘multiplexing’ or altered ‘multiple gene variants’ (26) (Table 1). Altered traits are quite diverse, covering, for example, storage management [114], root growth properties [63], flowering time [115,116], and improved yield [117]. Most of the studies **altering food and feed quality** (47) were performed on rice and tomato plants, but they also use many polyploid plants (18), such as camelina [78,118], potato [75,119] or wheat [74,81], which explains the increased number of studies falling under the category ‘multiple gene variants’. SDN-1 is mainly used to alter product quality, e.g., to alter the fatty acid composition [77,120] or sugar content [121] of the plants. The vast majority of studies generating **herbicide tolerant plants** used SDN-2 and SDN-3 applications (not shown here) and only a few used SDN-1 applications (3). Most studies targeted the ALS or EPSPS gene in order to induce herbicide tolerance. SDN-1 applications in plants for **enhanced breeding** were mostly performed on rice and maize plants. Some studies in this category can be considered to be basic research, frequently inducing haploidy [122,123,124] or male sterility [125,126,127]. Most of the studies using SDN-1 applications to change crop plants for **industrial utilization** altered multiple gene variants (7). In these studies, multiple alleles in polyploid crops [76,128,129,130], but also members of the BBL gene family in diploid tobacco [80] and multiple copies of the highly repetitive COMT gene [82,84], were altered. Multiplexing approaches were mostly used to generate plants with multiple altered traits (4) [92,131,132,133]. Plants in this category contain **multiple traits** that belong to different categories of market-oriented studies, such as a tomato plant altered using CRISPR/Cas9 for increased yield, modified product quality (modified lycopene content) and growth characteristics [92]. Thus, the tomato plant falls into two categories of market-orientated studies in plants, i.e., altered food and feed quality, and altered agronomic value. However, multiplexing in plants does not necessarily lead to plants with multiple traits, because single traits in plants are also very often dependent on multiple genes.

**Figure 2:**
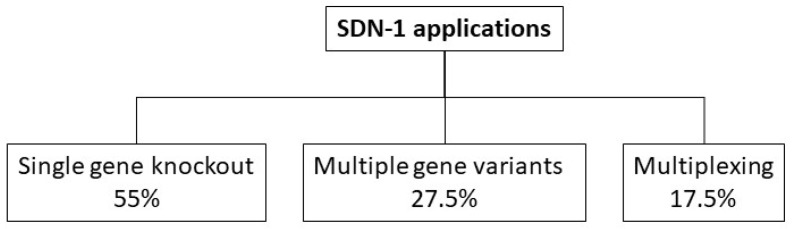
The outcome of the categorization for SDN-1 application types in crop plants is shown. In total, 98 studies used genome editing for ‘single gene knockouts’ in diploid plant species (i.e., 55% of all studies analyzed), 49 studies induced alterations in ‘multiple gene variants’ (i.e., 27.5% of all studies analyzed), and 31 studies used ‘multiplexing’ (i.e., 17.5% of all studies analyzed)-based on data from 1996 to June 2019.

The overall outcome of the categorization of SDN-1 application types is shown in Figure 2.

There were 98 studies that used SDN-1 for ‘single gene knockouts’ in diploid plant species and 49 studies induced alterations in ‘multiple gene variants’, including multiple alleles of a gene in a polyploid crop, multiple members of a gene family and multiple gene copies. Furthermore, 31 studies used ‘multiplexing’ to alter multiple, different target sites simultaneously or successively. Overall, there is the tendency to use genome editing for complex genetic alterations in order to alter more than two target sites in the genomes of plants.

#### 3.3.2. Development of Complex SDN-1 Applications over Time

**Figure 3:**
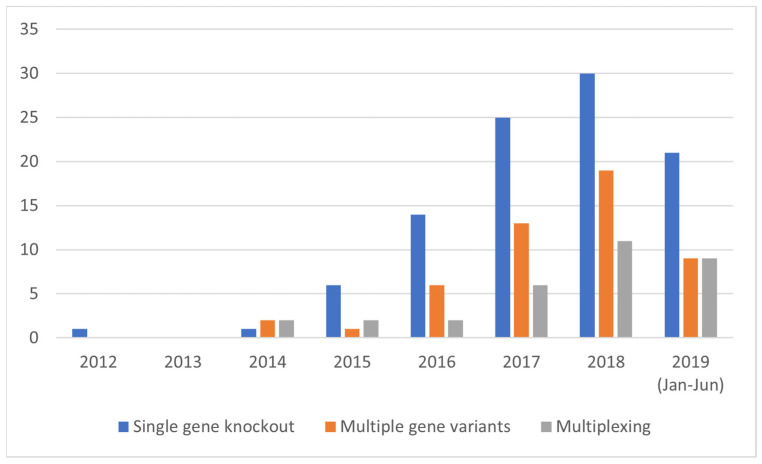
Distribution of studies using SDN-1 approaches (i.e., ‘single gene knockouts’, ‘multiple gene variants’, and ‘multiplexing’), between 2012 and June 2019 (data derived from [58]).

CRISPR/Cas9 was first applied in plants in August 2013 [134]. From then on, the number of publications describing CRISPR/Cas9 in plants increased continuously. Modrzejewski et al. [58] investigated market-oriented genome editing applications in plants within a timeframe from 1996 to June 2019. The focus of this current paper is on SDN-1 applications, which is why the identified relevant timeframe is between 2012 and 2019. The most frequently used technology, CRISPR/Cas, was used in 178 out of 217 studies between 2012 and June 2019 [58]. Figure 3 shows the distribution of SDN-1 application types between 2012 and June 2019. Studies that used genome editing techniques before 2012 were not considered here. All SDN-1 application types increased over time, with single gene knockouts being the most frequently application.

## 4. Regulation and Risk Assessment of SDN-1 Applications

The European Court of Justice ruled that all genome edited plants in the EU must be regulated under EU Directive No 2001/18/EC [28,135,136] and are required to undergo environmental as well as food and feed risk assessments [28,137,138]. Current GMO regulation thus covers both process- and product-based risks.

At present, there are ongoing discussions regarding the regulation of some genome edited plants (i.e., derived from targeted mutagenesis and cisgenesis), with one option being to solely focus on the end-product and its potentially new intended characteristics, while leaving aside unintended effects [29]. Another option would be to exempt SDN-1 applications from the directive [30].

The following section discusses why the risk assessment of plants altered with SDN-1 applications should address both process- and product-based aspects. Firstly, the relevant aspects for the process-based risks associated with SDNs, and particularly CRISPR/Cas, are summarized. Secondly, the relevance of the results from the categorization of SDN-1 application types for the regulation and risk assessment of SDN-1 applications are being discussed. Herein, it is differentiated between plants resulting from SDN-1 applications that carry traits already known from conventional breeding techniques and those that are so far unknown.

### 4.1. Generic Process-Based Risks

The main focus in the following section is on CRISPR/Cas, which is the most frequently used SDN in plant crops. Multiple studies have shown that CRISPR/Cas is able to induce unintended genomic alterations, such as off-target effects (unspecific cutting of CRISPR/Cas at unintended off-target sites), on-target effects (including large structural genomic changes such as translocations, large deletions, duplications or inversions), and integration of unintended DNA fragments at various parts of the genome [32,139,140,141,142,143,144,145,146,147,148,149,150,151,152,153,154,155,156,157]. These unintended alterations could potentially lead to a variety of unexpected effects: the integrity of a non-target gene may be compromised if its coding region has been cleaved unintentionally by CRISPR/Cas, which could lead to changes in the organisms’ metabolism, affecting its toxicity and allergenicity. Such effects are highly dependent on the genomic context within which such unintended alterations occur.

The current discussion about the potential of CRISPR/Cas is seen mainly in the context of the benefits of the technique rather than its generic risks. However, the generic risks associated with CRISPR/Cas arise equally from its potential, and can lead to new types of risks compared to previous genetic engineering applications: the ability of CRISPR/Cas to recognize and induce DSBs at all DNA regions carrying the same DNA sequence is highly relevant to risk assessment. If a plant contains several off-target sites—for example, in the DNA sequence of a gene within a gene family- then several or all of the genes in this gene family can be altered simultaneously. If an off-target site exists several times in a polyploid plant, off-target effects and other effects can occur at multiple sites of the genome. Therefore, CRISPR/Cas can modify an off-target region as often as gene variants (e.g., alleles or gene copies) with this DNA sequence are present in the genome in such plants.

SDNs induce alterations at the specific targeted genomic sites, which is the main difference to conventional breeding and induced mutagenesis. If a DSB induced by CRISPR/Cas is restored to its original state, the nuclease can recognize and cut the DNA sequence again. Thus, ultimately, the repair of DSBs induced by CRISPR/Cas is extremely error-prone [158,159]. There is also evidence that certain areas of the genome mutate less frequently than others, most likely due to the recruitment of the MMR by H3K36me3 and the activity of the MMR (see Section 2 and [37,38,39] for further information). Hence, if CRISPR/Cas induces a DSB in one of these genomic regions, it is very likely that it will ultimately induce a sequence alteration (whether intended or not), although presumably efficiency is reduced. Therefore, CRISPR/Cas also has the potential to induce unintended changes at off-target sites of the genome located within ‘protected’ genomic regions that have a naturally much lower mutation rate. Depending on the respective plant species and its ploidy level, not only one copy of an off-target site, but several or all copies can be altered simultaneously, though not necessarily in the same way. Furthermore, unintended alterations can be induced in areas of the genome that are mostly inaccessible to conventional breeding techniques (e.g., heterochromatin regions and in low recombinogenic genomic regions) [39,43,73,160].

Additionally, the intended alterations at the target sequence can induce unintended frameshift mutations or exon skipping, which can ultimately lead to the formation of new open reading frames [141,142,147,161]. Such unintended effects are highly relevant to complex changes, such as simultaneous alterations of several members of a gene family induced, for example, with CRISPR/Cas [80,81]. CRISPR/Cas induces an individual change at each individual target site and can generate new open reading frames. Ultimately, new mRNAs and corresponding proteins can be formed at each individual target site, which in turn may cause subsequent unintended effects. In addition, regulatory RNA species can also be formed differently.

One example to illustrate the generic risks of CRISPR/Cas is a wheat generated by Sanchez-Leon et. al. [81]; 45 α-gliadin genes were identified in a bread wheat cultivar. CRISPR/Cas9 was used to target a conserved region in the α-gliadin genes using a gRNA. In one line, 35 different genes of the 45 α-gliadin genes were altered simultaneously. CRISPR/Cas induced different alterations at each individual target site as indicated by different deletion or insertion types found at the target locus of the gRNA ‘sgAlpha-2′ (see Figure 1b and Figure 1c respectively in [81]). The same study was also listed as an example by the European Food Safety Authority (EFSA) in its recent scientific opinion on the ‘Evaluation of existing guidelines for their adequacy for the molecular characterization and ERA of GM plants obtained through synthetic biology’ regarding the analysis of complex alterations induced by CRISPR/Cas [57]. EFSA assigned the wheat with a decreased α-gliadin content to synthetic biology [57]. Synthetic biology is a more advanced form of genetic engineering with an increased depth of intervention. According to EFSA, their case study shows that a strategy is needed to identify the type of alteration and position in each individual gene to prevent the accumulation of any unintended peptide fragments [57]. Such analyses are of major importance for risk assessment, especially when considering SDN-1 applications with a higher level of complexity and/or depth of intervention (see Section 3).

There are further causes of unintended changes which may be associated with specific risks: in most of the 178 market-oriented studies considered here, classical (or first generation) genetic engineering techniques were routinely used to transform and randomly integrate the DNA for the formation of the CRISPR/Cas components into the plant genomes. Over 90% of the studies, used either *Agrobacterium* transformation or particle bombardment (151 out of 178 studies used *Agrobacterium* transformation and 10 out of 178 studies used particle bombardment). These classical techniques are still regularly used due to existing and well-established protocols for plant transformation.

Old genetic engineering techniques are known to induce unintended alterations in plant genomes [162,163,164,165,166,167,168]. This has been investigated, for example, in soybeans [152,169], rice [156] and arabidopsis [164], which were transformed by *Agrobacterium tumefaciens*. These studies show that the transgenic DNA and vector backbone can be inserted once, multiple times, fragmentarily and at different random locations in the genome. If additional transgenic DNA remains unnoticed in the genome, unintended changes can occur in subsequent generations. DNA sequences can also be restructured during the process of *A. tumefaciens* transformation [162,164], including large deletions, translocations, or inversions [164,167,170,171,172], and epigenetic markers can be altered at the integration site(s) [164].

Unintended effects have also been described for particle bombardment [165,167,173,174]. The integration of the transgenic DNA is an untargeted, random process that can result in multiple integration events [167]. It has also been shown that insertions and rearrangements of the DNA can occur during particle bombardment [167]. Therefore, crop plants altered using SDNs in combination with classical genetic engineering techniques should be assessed for unintended changes if they are to be placed on the market.

Some risks associated with SDNs might be similar to those assessed in previous applications of genetic engineering in plants. However, new types of risks also have to be considered. A systematic analysis of plants derived from SDN-1 applications is needed in order to investigate different forms of unintended alterations (i.e., off-target effects, on-target-effects, integration of additional DNA, and production of new gene products) induced by SDNs. These techniques have the potential to induce complex unintended alterations due to their mode-of-action (e.g., inducing different alterations at multiple genomic sites with the same sequences). This needs to be acknowledged and addressed in hazard identification and risk assessment. The entire technical process used to generate a particular trait should be considered in each case when carrying out case-by-case risk assessments. The way in which CRISPR/Cas was delivered into the plant cells also needs to be taken into account, i.e., whether in the form of DNA, mRNA, or ribonucleoproteins, and what effects might result from these processes.

### 4.2. Product-Based Risks

Systematic reviews of the scientific literature have shown that SDN-1 applications are being used in crop plants to develop a broad range of traits [12,13]. It has also been shown that a wide range of plant species is being altered using SDNs. Some of these traits are already known from previous breeding, while others are novel.

The following section examines considerations for the risk assessment of end-products derived from SDN-1 with either known or currently unknown traits.

#### 4.2.1. Traits That Are Known from Previous Breeding

SDN-1 can be used to generate traits that are already known from conventional breeding. There is currently some discussion around less rigorous requirements for the risk assessment of such plants [29,175]. However, if it is assumed that the traits of SDN-1 plants, i.e., their performance and safety status, can be equated with those of conventional breeding, this must be firmly established by appropriate risk assessment, including molecular characterization, phenotypic characterization, and metabolic analysis. In most of the studies analyzed in this paper, there was no explicit investigation of whether a particular SDN-1 plant can be equated with a comparable variety from conventional breeding. Some studies allow for such a direct comparison of the outcome of induced mutagenesis and SDN-1, e.g., powdery mildew-resistant wheat [176,177]. Loss-of-function alleles of the *Mildew resistance locus o* (*Mlo*) gene in barley confer powdery mildew resistance. The redundancy of the *mlo* genes in the wheat genome (*TaMlo-A1*, *TaMlo-B1* and *TaMlo-D1*) prevented the analysis of whether mutations of all three *mlo* genes might confer resistance to fungal powdery mildew, a trait not found in natural populations [177]. In one study, the *mlo* gene was targeted in hexaploid wheat by TALENs [177] and in another study by chemical mutagenesis using EMS treatment followed by a TILLING analysis [176], which allowed direct identification of mutations in a specific gene region, to generate resistance to powdery mildew. Plants obtained by EMS/TILLING are exempt from EU GMO legislation, based on the long-termed use of mutagenesis. TALENs mediated different alterations in all three homoeoalleles of *mlo* in wheat, ultimately enabling the generation of homozygous *mlo* mutant wheat plants. The simultaneous alteration of the three homoeoalleles conferred a broad-spectrum resistance to powdery mildew in these lines [177]. The EMS/TILLING approach also successively generated homozygous triple mutant lines which showed enhanced but incomplete resistance to powdery mildew disease, indicating that only partial loss-of-function alleles were generated in these lines [176].

The TALENs approach most likely generated *mlo* null mutations, which resulted in the production of a nonfunctional protein or no protein at all. In contrast, the EMS/TILLING approach generated *mlo* mutant lines, which can be considered as a gene knockdown of the *mlo* genes. The EMS-mutant *mlo* wheat most likely generates fewer gene products, thus explaining the moderate resistance to powdery mildew disease. The example of the two powdery mildew-resistant wheats highlights a major difference between SDNs and conventional breeding techniques, i.e., the genomic context and the dimension of the changes induced by SDNs. This means that the exact position where an alteration in a gene is induced matters and determines the respective outcome, as well as the numbers of changes made. SDNs enable the generation and combination of alterations at precise positions in the genome, whereas with conventional breeding techniques, random mutations are induced. In some cases, SDNs can induce alterations in genomic regions where no mutations have ever previously been described (Section 4.2.2).

In addition, and importantly, pleiotropic effects were described in the TALEN wheat (i.e., leaf chlorosis under growth conditions), [177] which were not observed in the TILLING mutants compared to the wildtype [176]. This indicates that, even though the same trait was altered, the end-products of the different techniques can differ significantly. Thus, there is a necessity to specify how exactly the equivalence of the two types of plants can be shown for regulation.

Furthermore, plants altered with SDN-1 which contain traits that are known from cultivated varieties, but are expressed in a new genetic background, cannot be equated to their conventional or natural counterparts, as the corresponding target gene(s) might have divergent functions or interactions in different species. De novo domesticated plants generated using CRISPR/Cas9 are interesting examples in that regard [92,132,178]. For de novo domestication, CRISPR/Cas9 is used to alter the genomes of wild species in such a way that some of their genes are modified to resemble domesticated ones. Such de novo domesticated plants still have some properties from wild species that have been lost during plant breeding. These traits are combined with domestication traits, for example, by multiplexing in wild species [92] that have proven to be important for cultivation. For example, already existing genetic variations from the cultivated diploid rice species, *Oryza sativa*, were introduced into the tetraploid wild rice species, *Oryza alta*, using CRISPR/Cas9 for multiple SDN-1 applications. Multiple genes were altered in *O. alta* in order to generate agronomically relevant traits, including seed shattering, awn length, plant height, grain length, stem thickness, and heading date, all of which are present in cultivated *O. sativa* [178]. Comprehensive environmental and health risk assessments will be needed to ensure that no effects with negative impacts have occurred.

Risk assessments also need to be carried out in order to address combinatorial and cumulative effects of multiple traits combined. If single traits known from previous breeding or from natural variations are combined in one plant by SDN-1 applications, this can result in a plant with new, previously unknown genetic combinations. Examples from the literature screening include multiplexing approaches that enable the modification of different target genes simultaneously [89,178,179,180] or successively [93].

#### 4.2.2. Traits Previously Unknown in Conventional Breeding

SDNs can be used to generate traits unknown in previous conventional breeding [175,181,182]. Examples of new traits present in each of the categories are shown in Table 1, indicating that new genetic combinations can result from all types of SDN-1 alterations [61,85,92]. In this context, it is important to discuss ways of establishing whether a genetic combination and corresponding traits can be considered to be already in existence. The findings of this publication could be used to facilitate a subsequent, more comprehensive analysis, investigating which genetic alterations induced by SDN-1 applications are already present in the natural gene pool of plants and previously obtained from conventional breeding. The pangenomes of crops (i.e., the entire gene set of all strains of a species) could serve as a reference for naturally occurring genetic variants and therefore depict the entire genetic diversity within a species in order to identify novel genotypes. The pangenomes of major crops that are frequently altered by genome editing, such as rice, tomato, and wheat plants have already been published [183,184,185]. More data is necessary to generate reliable pangenomes of more crops with complex genomes, as well as for understudied plants such as orphan crops [186].

As mentioned above, CRISPR/Cas can be used to induce alterations in those parts of the genome that correlate with a lower mutation rate (i.e., where fewer de novo mutations occur) (see Section 2) [37,38,39]. The first CRISPR/Cas-product released for consumption in Japan, a tomato with an increased GABA-content, contains genetic alterations which were so far not achievable using chemical mutagenesis [61]. It is reasonable to suppose that mutations in this particular part of the target genes are naturally blocked by the activity of the MMR. More research is needed to a) correlate datasets, such as those generated by Monroe et al. [37], in various crops with CRISPR/Cas induced alterations and b) to specifically investigate how efficiently CRISPR/Cas can induce alterations in genomic regions that correlate with increased MMR activity. It is very likely that many genetic alterations in studies which fall under the category of SDN-1 application type ‘single gene knockout’, are not previously known from conventional breeding, e.g., the tomato with an increased GABA content [61].

## 5. Discussion

This paper describes and investigates aspects relevant to the regulation of crop plants altered using SDN-1 applications. It shows that SDN-1 applications cannot be generally exempt from GMO regulation in the EU.

The dataset generated by Modrzejewski et al. [58] was analyzed in this context with regards to the SDN-1 application types used in market-oriented crop plants in order to gain a better understanding of the complexity of these alterations. SDN-1 application types were classified into three different categories, i.e., ‘single gene knockouts’, altering ‘multiple gene variants’, and ‘multiplexing’. The majority of SDN-1 applications are single gene knockouts (98 out of 178 studies analyzed) (see Figure 2). Many studies fall under the categories of ‘multiple gene variants’ (in total 49 out of 178) and ‘multiplexing’ (31 out of 178 studies), indicating that SDN-1 applications are also being used to induce complex and multiple alterations in crop plants.

Plants altered with SDN-1 applications can contain genetic alterations that either result in novel and unknown traits, or in already known traits from conventional breeding, or natural genetic variations. Already known traits can be combined with other known or unknown traits—presumably resulting in unknown genetic combinations -, or can be induced in genetic backgrounds of plant varieties, where the specific genetic alterations are so far not present in conventionally bred lines. It is therefore important that the final results of SDN-1 applications be precisely differentiated and characterized. EFSA did not specify in their published opinion [187] how different combinations of known traits in plants derived from SDN-1 applications should be assessed. The crossing of plants derived from different SDN-1 applications may lead to unintended combinatorial effects in the progeny. Therefore, SDN-1 plants obtained from further crossings after market approval should undergo risk assessment, in the same way as stacked events derived from previous methods of genetic engineering (see [158]).

In cases where two plants of the same species derived from SDN-1 applications and conventional breeding techniques both have the same trait, it needs to be specified how the equivalence of the two types of plants is to be proven for regulation.

SDN-1 applications allow modifications ranging from ‘simple’ alterations that are well known in a particular species to complex combinations of several alterations affecting different traits. A general assumption that SDN-1 alterations are identical to naturally occurring mutations or induced mutations, and are therefore just as safe, cannot be made. The specific risk of a product has to be assessed individually and general exemptions of SDN-1 applications from the GMO regulation cannot be justified.

It is also likely that many ‘simple’ alterations, such as single gene knockouts will give rise to so far unknown traits (for that species) as, for example, in the genome edited tomato with an increased GABA content [61]. Further development and improvement of pangenomes representing naturally existing genetic variants and those derived from conventional breeding could perhaps facilitate a differentiation supported by the rapidly increasing knowledge on plant genomics.. However, even where a gene variation is found in a pangenome, the respective genetic background needs to be considered, especially when the specific genetic alterations are not present in conventionally bred lines at this point.

A recently published review discusses different biosafety aspects that need to be considered for the regulation and risk assessment of SDN-1 applications, including, e.g., the speed of their development, the depth of intervention, or the number of genetic changes [36]. The authors conclude that the principles and the case-specific approach provided in the current framework for GMOs is appropriate for SDN applications and that, from a biosafety perspective, whole classes of genome editing applications (especially SDN-1) cannot be excluded. This outcome is in line with the findings presented here.

In addition to the risks associated with the end-product (i.e., unintended effects that could impact the health of consumers and the environment), the risks associated with the multistep process of applying SDN-1 in plant cells also need to be considered. Many publications have already shown that CRISPR/Cas induces off-target and unintended on-target effects, in addition to unintended frameshift mutations that can generate new open reading frames causing the formation of new gene products. It has also been shown that DNA fragments from different sources can be unintentionally integrated at the target site or elsewhere in the genome. Moreover, the majority of SDN-1 applications still rely on classic transformation techniques or other techniques to deliver the molecular tools which are also known to induce unintended alterations. Thus, the risk assessment of SDN-1 plants will also need to consider such unintended alterations if they are used.

The potential of the technology and its significance for unintended changes in the genome and potential new types of risks presented here should be considered in the current debate on the regulation of plants derived from SDN-1 applications. More precisely, the impression often given is that unintended changes induced by CRISPR/Cas are identical to random mutations induced in conventional breeding techniques. The differences are the quality and the quantity of unintended changes induced by CRISPR/Cas: all copies of off-target sites that have the same or sufficiently similar DNA sequence can be altered (e.g., all alleles or all gene copies). Furthermore, off-target sites in parts of the genome that are less accessible for conventional breeding can be unintentionally altered. Regarding the changes at the target site(s), there needs to be acknowledgement that, at each individual target site present in the genome, an individual alteration is induced by CRISPR/Cas which can lead to the formation of new peptides or proteins or even frame shifts causing nonsense sequence changes. The application of SDN-1 in plants requires knowledge of the target site(s) which can be used for the risk assessment of the variety of target site alterations, to identify all off-target sites and to check for unintended alterations.

## 6. Conclusions

In summary, this review here shows that about half of the market-oriented plants developed by SDN-1 applications contain complex alterations in their genome (i.e., altering multiple gene variants or using multiplexing). It also illustrates that data on both the process- and the end-product are needed for a case-by-case risk assessment of genome edited plants. The broad range of genetic alterations and their corresponding traits reflects how diverse and complex the requirements are for such a risk assessment.

At present, the existing EU GMO legislation [136] seems to be best suited for the regulation and risk assessment of genome edited plants, including SDN-1 applications. Some adjustments will be needed to guide data generation and data assessment. This necessary guidance can be developed within the current regulatory system. As required presently by EFSA, specific datasets need to be submitted for the assessment of different traits (such as insect toxicity, herbicide tolerance, intended drought tolerance or changes in oil composition). This also impacts the design of the field trials, the required toxicity studies and the overall risk assessment. Therefore, the existing system is likely to provide the necessary flexibility also for SDN-1 applications.

## Figures and Tables

**Table 1:** Results from the categorization of SDN-1 application types in plants according to trait or purpose (between 1996 and July 2019). Shown are the number of studies that used SDN-1 applications to generate either ‘single gene knockouts’, alter ‘multiple gene variants’, or apply ‘multiplexing’, per category of market-orientated applications and in total.

Category of Market-Orientated Applications	Single Gene Knockouts	Multiple Gene Variants	Multiplexing	Total
Abiotic stress resistance	3	2	1	6
Biotic stress resistance	15	9	4	28
Agronomic value	35	11	15	61
Food and feed quality	24	18	5	47
Herbicide tolerant plants	2	-	1	3
Enhanced breeding	17	2	1	20
Industrial utilization	1	7	-	8
Multiple traits	1	-	4	5
In total	98	49	31	178

## Data Availability

Not applicable.
